# Chicken Cartilage-Derived Carbon for Efficient Xylene Removal

**DOI:** 10.3390/ijms241310868

**Published:** 2023-06-29

**Authors:** Joanna Dobrzyńska, Zuzana Jankovská, Lenka Matějová

**Affiliations:** 1 Department of Analytical Chemistry, Institute of Chemical Sciences, Faculty of Chemistry, Maria Curie-Sklodowska University, M. C. Sklodowska Sq. 3, 20-031 Lublin, Poland; 2 Institute of Environmental Technology, Centre for Energy and Environmental Technologies, VŠB—Technical University of Ostrava, 17. listopadu 15/2172, 708 00 Ostrava, Czech Republic; zuzana.jankovska@vsb.cz (Z.J.); lenka.matejova@vsb.cz (L.M.)

**Keywords:** waste animal bones, microwave pyrolysis, microporous carbon adsorbent, adsorption, volatile organic compounds (VOC)

## Abstract

Chicken cartilage was used for the first time as a raw material for the microwave-assisted synthesis of biochar and activated carbon. Various microwave absorbers, i.e., commercial active carbon, scrap tyres, silicon carbide, and chicken bone-derived biochar, as well as various microwave powers, were tested for their effect on the rate of pyrolysis and the type of products formed. Biochars synthesised under 400 W in the presence of scrap tyres and chicken bone-derived biochar were activated with KOH and K_2_CO_3_ with detergent to produce activated carbon with a highly developed porous structure that would be able to effectively adsorb xylene vapours. All carbons were thoroughly characterised (infrared spectroscopy, X-ray fluorescence spectrometry, nitrogen adsorption/desorption, Raman spectroscopy, proximate and ultimate analysis) and tested as xylene sorbents in dynamic systems. It was found that the activation causes an increase of up to 1042 m^2^·g^−1^ in the specific surface area, which ensures the sorption capacity of xylene about 300 mg·g^−1^. Studies of the composition of biogas emitted during pyrolysis revealed that particularly valuable gaseous products are formed when pyrolysis is carried out in the presence of silicon carbide as a microwave absorber.

## 1. Introduction

Volatile organic compounds (VOC) are organic substances with a characteristically low boiling point, high vapour pressure, and strong reactivity at 25 °C. VOC are extremely dangerous for both human health and the environment [1]. Xylenes are one of the most crucial aromatic VOC obtained from light coal tar fractions, from which the mixture of all isomers is distilled [2]. The mixture of three isomeric xylene forms (*ortho-, metha-, and para-*) is applied as solvent and thinner in inks, paints, cleaning agents, and varnishes [3]. Additionally, *o*-xylene is used for the manufacture of phthalic anhydride, *m*-xylene for the production of plastics and dyes, and *p*-xylene for the synthesis of terephthalic acid [4]. A small number of xylenes are also detected in gasoline and aeroplane fuel. Xylenes pose a serious threat to human beings and the environment. They are cancerogenic and harmful to the lungs, kidneys, liver, and nervous system. Xylenes may also cause throat and nasal irritation, gastric discomfort, nausea, and vomiting [5]. They can be introduced into the body by inhalation, orally, and, sometimes, if in a small amount, by the dermal route [5].

In order to reduce the exposure of people working in an atmosphere containing xylenes to the negative effects of these compounds, various methods of xylene removal from the air have been developed. Among the methods of xylene removal such as condensation [6], catalytical oxidation [7], or biofiltration [8], the most popular one seems to be its adsorption onto solid sorbents [9,10]. For this purpose, carbon materials such as activated carbon [11], biochars [12], nanotubes (CNTs) [3], or graphene [13] are usually chosen due to their extensive porous structure and relatively high adsorption affinity towards non-polar xylene. 

In recent years, a lot of research has been carried out on the synthesis of biochar from waste materials produced in broadly understood agriculture. Abundant and inexpensive agricultural residues contain a lot of carbon and can be easily converted into biochar by pyrolysis [14], hydrothermal carbonization [15], or gasification [16]. The synthesis of biochar not only makes it possible to obtain a sorbent useful for volatile organic compounds adsorption [16,17] but also contributes to the sequestration of carbon dioxide from the atmosphere, thus reducing the greenhouse effect. 

The currently used biochar synthesis methods assume that the raw material is heated and carbonised from its outer space in inert conditions; however, this approach requires quite a lot of energy and a long reaction time [16]. To minimise the carbon footprint associated with pyrolysis, solar energy has been used for the synthesis of biochar for several years. Owing to the use of optically reflective concentration devices, it is possible to carry out this ecological type of pyrolysis; however, this process, like conventional pyrolysis based on heat convection and conduction, requires a long reaction time to reach the desired temperature [18]. A significant reduction in the time required to convert biomass into biochar while reducing energy consumption can be achieved by microwave-assisted pyrolysis, in which non-contact conversion of microwave energy into thermal energy occurs [19]. The microwave energy is adsorbed by biomass, and microwave pyrolysis can be initiated at a temperature of around 600 °C [20]. The intermediate products of microwave-assisted pyrolysis of plant biomass differ from those obtained in conventional processes, probably because of the simultaneous degradation of hemicelluloses and lignin under microwaves in contrast to their sequential decomposition during heating [21]. In the case of biomass-based materials that poorly absorb microwaves due to their low dielectric properties, the addition of the absorber is necessary to initiate the pyrolysis. This role may be played by biochar, soot, activated carbon, or silicon carbide. Owing to the application of microwave pyrolysis, rapid, volumetric, selective, and uniform heating of biomass can be obtained [22]. So far, microwave-assisted pyrolysis has been mainly used to synthesise biochar and char from plant-derived materials (e.g., pecan nutshells, woody biomass, sugarcane bagasse, oat hull), sludges, and tyres [19,20,21]. There are no reports in the literature on the use of microwaves to synthesise biochar from waste from the animal industry. Based on the results presented in [12] concerning the application of biochar conventionally synthesised from animal bone and shells for the effective removal of VOC from the gas phase, we undertook the task of synthesizing biochar and activated carbon from chicken cartilages by microwave-assisted pyrolysis. By using this approach, the time and energy expenditure necessary to obtain a carbon that effectively removes xylene will be reduced, and chicken cartilage, which is produced in large quantities in European countries, will also be managed.

Cartilages have not been reported as a raw material for biochar or activated carbon synthesis; however, there are numerous reports on the use of bones for this purpose. Chicken bones are an excellent raw material for the production of carbon materials and are used as sorbents for organic and inorganic pollutants from the liquid and gas phases [12,23,24,25]. Due to the hybrid organic-inorganic nature and the spatial structure of bones, the biochar obtained from them is characterised by a large surface area, often exceeding 1000 m^2^·g^−1^ [26]. In addition to organic matter, bones contain calcium oxide (CaO) and hydroxyapatite (Ca_5_(OH)(PO_4_)_3_), which have been found to be the template of mesopores. The factor contributing to the increase in porosity is also carbon dioxide and water vapor, which are released during the thermal decomposition of hydroxyapatite and CaCO_3_. Additional modification by KOH of biochar obtained from bone increases the already large surface area to values exceeding 2000 m^2^·g^−1^ [26]. The large specific surface area and the presence of micropores in carbon generated by the activator favour the adsorption of VOC. 

In addition to bones, the slaughter industry also generates huge amounts of cartilage, which can be converted to biochar or activated carbon. This material is structurally different from bone and consists mainly of glycosaminoglycans, proteoglycans, collagen fibres, and elastin [27]. Thus, cartilage does not contain significant amounts of calcium carbonate or hydroxyapatite, whose thermal decomposition would result in significant porosity in the biochar formed during microwave-assisted pyrolysis. Therefore, in order to obtain carbon with a large specific surface, the synthesis should be carried out in the presence of activators, the addition of which will result in the formation of an extensive porous structure. 

In this work, microwave-assisted synthesis of a series of carbons from chicken cartilage was performed and described for the first time. The effect of microwave power (200 and 400 W) on the physicochemical properties of the obtained biochar was investigated. We monitored how the type of microwave ray absorber (silicon carbide, active carbon, chicken bone-derived biochar, scrap tyres) and the type of activator (KOH, K_2_CO_3_) affect the properties of carbon. The influence of the addition of a commonly available surfactant, i.e., dish soap, to the mixture of activator, absorber, and feedstock on the properties of the obtained carbon material was also studied. Finally, the obtained carbons were tested for xylene removal.

Based on the obtained results, the most appropriate microwave absorber, microwave power, and activator are chosen, which enable the synthesis of highly porous carbon from chicken cartilage. It is expected that the carbon obtained under optimal conditions will effectively adsorb xylene and, thanks to the possibility of its regeneration, will be able to find practical application for air filtration.

## 2. Results

### 2.1. Characterisation of Raw Cartilage

Based on the cartilage analysis results presented in Table 1 and Table 2, it can be concluded that, from a chemical point of view, proteins and oil are the main constituents of the chicken cartilage. The content of proteins by weight is 51.1%, whereas the oil content is 24.1%. Sugar (starch + invert) content is 0.45%, while fibber is only 0.3%. The moisture content of the cartilage is 2.8%, which is comparable to that obtained for nonactivated biochar. Raw cartilage is characterised by a very high ratio of volatile compounds (79.5%). Due to the high proportion of volatile compounds, the fixed carbon content is only 11.0% and the ash content is 6.6%. A high content of volatile compounds means that the storage or burning of unprocessed cartilage may contribute to greenhouse gas emissions. Based on the XRF analysis results, it can be concluded that the main inorganic constituents of raw cartilages are calcium (0.67%), sulphur (0.65%), phosphorus (0.54%), potassium (0.52%), and chlorine (0.17%).

The surface of raw cartilage is not developed as cartilage is not a porous material. The value of the S_BET_ was only 0.96 m^2^·g^−1^ and V_net_ was 0.87 mm_liq_^3^·g^−1^.

### 2.2. The Influence of Microwave Power and Microwave Absorber on Pyrolysis Rate and Product Yields

**Figure 1:**
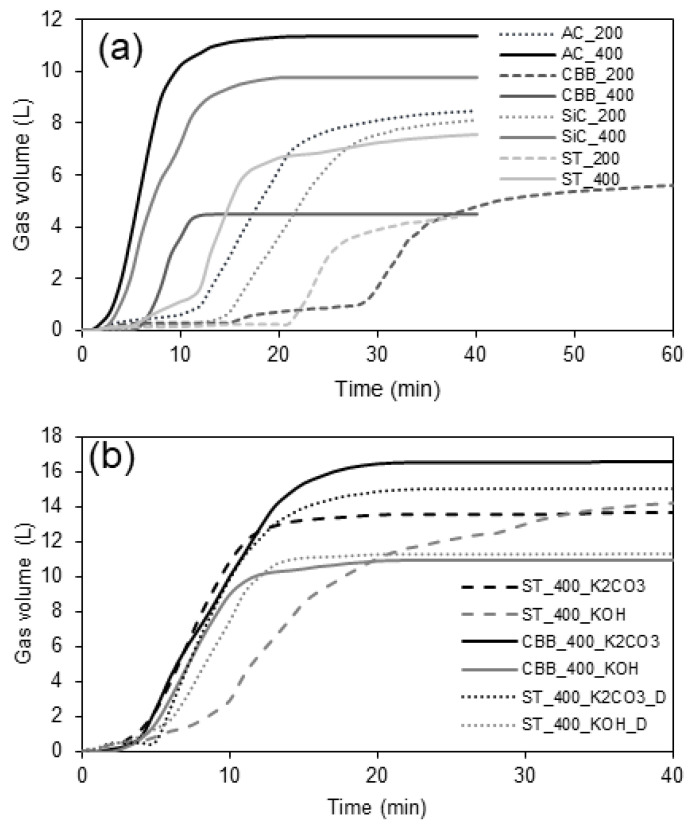
Total amount of gas evolved during pyrolysis process carried out (**a**) without activator and (**b**) with activator.

Based on the relations shown in Figure 1, it was found that the rate of pyrolysis and hence the rate of gas evolution from decomposing cartilage depend on the power of the microwaves and the type of microwave absorber. For all studied systems, which are presented in Table 3, regardless of the type of microwave (MW) absorber (active carbon—denoted as AC; scrap tyres—denoted as ST; chicken bone-derived biochar—denoted as CBB; silicon carbide—denoted as SiC); pyrolysis is faster by approx. 10 min under 400 W than 200 W power. Regarding the presence of an activator, a higher amount of gas is produced in the presence of K_2_CO_3_ (12–18 L) than in the presence of KOH (10–13 L). However, there is no point in increasing the power above 400 W because for higher powers, the large volume of gases evolving per unit time and the high pressure cause the reaction system to become unsealed. Therefore, in order to ensure the tightness of the system and, at the same time, to minimise the duration of pyrolysis, the optimal microwave power seems to be 400 W. 

As mentioned above, AC, ST, CBB, and SiC were added to the biomass and tested as MW absorbers. It was found that a properly selected MW absorber can significantly increase the rate of pyrolysis. When a microwave power of 400 W was applied, the slowest pyrolysis was observed for reaction mixtures containing ST as MW absorbers. Tyres contain a certain amount of soot that is able to absorb microwave radiation; however, the amount of soot contained in 2 g of tyres is too small to lead to the rapid pyrolysis of 50 g of cartilage. The fastest pyrolysis occurs when AC is used; this material, due to the very small size of the particles, effectively mixes with the cartilages and sticks to their surface, allowing for effective heating of the entire volume of biomass. Taking into account only the influence of the absorber on the rate of pyrolysis, the most advantageous seems to be the application of AC, SiC, or CBB for this purpose, because in the case of these three absorbers, the time after which gas is no longer released from the biomass is the shortest, i.e., only around 10–12 min. When ST is used, it can take up to 20 min.

In terms of the amount of gas produced, the degree of influence of the absorber can be divided into three groups: AC_400, SiC_400 (total amount of gas ~10 L) > AC_200, SiC_200, ST_400 (total amount of gas ~8 L) > CBB_400, ST_200, and CBB_200 (total amount of gas ~4 L). However, apart from the rate of pyrolysis, the mass and composition of the biogas and biooil produced are the most important parameters determining the selection of a specific microwave absorber, in addition to the physicochemical properties, mainly the specific surface area and the degree of graphitization of the obtained biochar. When the overarching goal of pyrolysis is to obtain biochar/activated carbon that provides high adsorption affinity to xylene, high graphitization, a high specific surface area, and a high meso- and micropore volume are expected from it.

**Figure 2:**
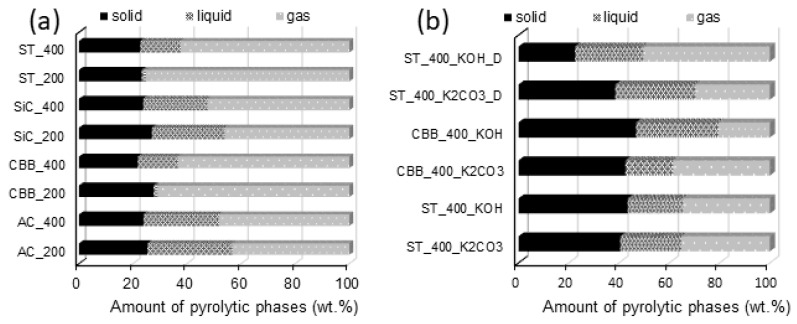
Mass balance of individual pyrolytic phases from cartilage pyrolysis (**a**) at 200 and 400 W for 40 min without activator and (**b**) at 400 W for 40 min with activators.

The relative yields of particular pyrolysis products depend on the type of raw material used for the synthesis, the type of microwave absorber, the composition of the reaction mixture (the presence or absence of activators), the construction of the reactor, the pyrolysis temperature, and the heating rate. At first glance, Figure 2 shows that the addition of an activator (KOH or K_2_CO_3_) to the reaction mixture during MW-assisted pyrolysis of cartilage results in a two-fold increase in carbon synthesis efficiency. This is because the solid product synthesised from cartilage in the presence of KOH or K_2_CO_3_ contains a significant amount of activator residues, i.e., mainly potassium (present in the carbon before washing). In the case of using KOH as an activator (ST_400_KOH and CBB_400_KOH), the yield of the solid product is around 3% higher than when the activation was carried out with K_2_CO_3_. This relationship is probably due to the higher molar fraction of potassium in KOH than in K_2_CO_3_. It can therefore be assumed that a significant part of the mass of the solid product (before washing with acids) in activated carbons is potassium [28].

It should be noted that in the case of the ST_400_KOH_D and ST_400_K_2_CO_3__D activated carbons, the percentage of the solid phase is low, which is caused by the transfer of a significant amount of cartilage from the reaction vessel to the pipe connecting the reactor with the cooler. In the reaction systems containing the addition of 10 mL of water and two drops of detergent, foam has been created. The volume of foam was especially large during the synthesis of ST_400_KOH_D because of the collagen that makes up cartilage, which reacted with KOH to form foam [29]. When the foam column was higher, more raw material particles were removed from the reactor and the mass of the final activated carbon was lower—the mass of ST_400_KOH_D was the lowest of all activated carbons.

Comparing the mass balance relationships obtained for syntheses carried out without the addition of the activator, it can be concluded that, regardless of the type of MW absorber, the use of higher MW power (400 W) results in the slightly (~4 wt.%) lower efficiency of carbon synthesis. In reactors heated with 400 W of power, a higher temperature is reached than in reactors heated with 200 W of power, favouring the decomposition of biomass into simple compounds of high volatility. Therefore, high microwave power promotes the decomposition of the solid and its transformation into volatile compounds, from which biooil and biogas are obtained [30].

It was found that in systems without activators, the application of CBB or ST as MW absorbers is conducive to obtaining a gaseous product (yield in the range of 63–75 wt.%), while for other MW absorbers the yields of biogas do not exceed 52 wt.%. For the studied reaction systems, there is no unequivocal relationship between the power of the microwaves and the yield of biogas and biooil. In the case of the CBB_200 and ST_200 systems, for which the pyrolysis reaction proceeded very slowly due to the low biomass heating rate, the amount of collected biooil was very small. This was probably due to the very slow flow of volatile compounds through the cooler. Compounds that remained at a low temperature for a relatively long time condensed not only to a liquid but also to a solid, which was collected at the end of the condenser instead of dripping into the liquid phase receiver. In instances where the same MW absorbers (CBB and ST) were used but with a higher microwave power (400 W), the pyrolysis reaction ran faster, so the volume of gases released per unit of time was greater. This resulted in their shorter stay in the cooler and enabled their collection in the form of a liquid. In these systems, the residence time in the cooler was too short for the liquid product to reach freezing point.

### 2.3. Characterisation of Carbon

#### 2.3.1. Structural Parameters of Activated Carbon and Biochar and Graphitisation

**Figure 3:**
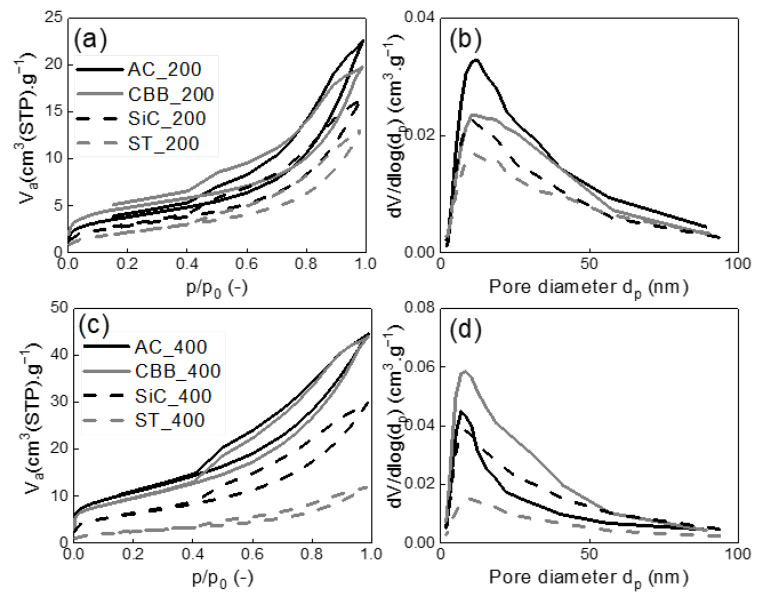
Measured nitrogen adsorption-desorption isotherms at 77 K (**a**,**c**), evaluated mesopore-macropore-size distributions (**b**,**d**) of produced biochar prepared by microwave pyrolysis of cartilage at 200 W (**a**,**b**) and 400 W (**c**,**d**) with different microwave adsorber.

**Figure 4:**
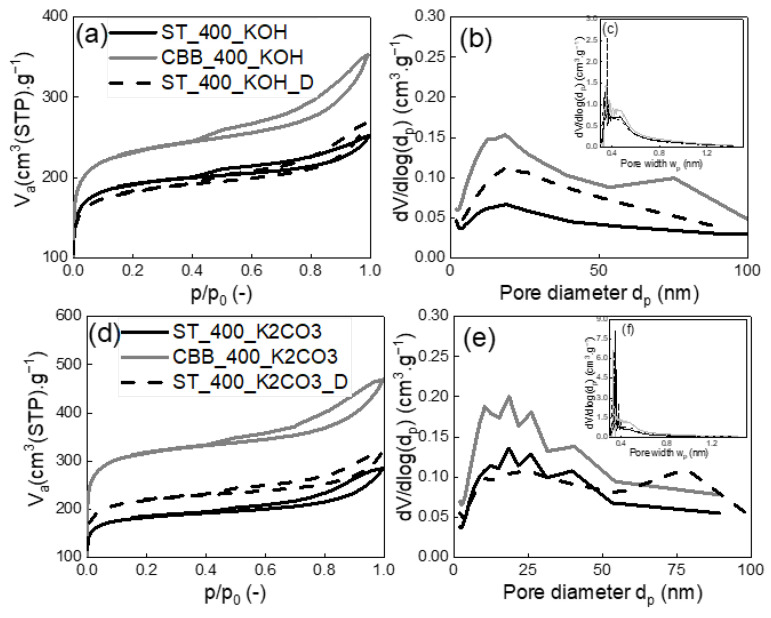
Measured nitrogen adsorption-desorption isotherms at 77 K (**a**,**d**), evaluated mesopore-macropore-size distributions (**b**,**e**) and evaluated micropore-size distributions (**c**,**f**) of produced activated carbon prepared by microwave pyrolysis of cartilage at 400 W and activated by KOH (**a**–**c**) and K_2_CO_3_ (**d**–**f**).

In order to determine the structural parameters of the synthesised carbons, nitrogen adsorption/desorption isotherms were measured and evaluated. The isotherms are shown in Figure 3 and Figure 4, while the structural parameters calculated based on the nitrogen adsorption/desorption data and I_D_/I_G_ ratio evaluated based on Raman spectra are presented in Table 4. 

For the biochar synthesised without the addition of surface activators, the unequivocal influence of the applied microwave power on the value of the specific surface area is observed. Regardless of the type of MW absorber, the pyrolysis under 400 W created biochar with a higher specific surface area than under 200 W. In the presence of AC, CBB, and SiC acting as MW absorbers, the specific surface area obtained under 400 W was approximately two times higher (23–39 m^2^·g^−1^) than that obtained under 200 W (11–17 m^2^·g^−1^). When ST is applied as the MW absorber, the increase in microwave power from 200 to 400 W caused only around a 10% increase in the specific surface area of the solid product. The highest surface areas of 39.1 and 34.5 m^2^·g^−1^ were obtained for AC_400 and CBB_400, respectively. Much lower values were obtained for SiC_400—22.8 and ST_400—9.3 m^2^·g^−1^. The specific surface area values correlate with the pore volumes. As presented in Figure 3b,d, all biochar pores with a diameter of about 12 nm dominate. V_net_ is relatively small and does not exceed 70 mm^3^·g^−1^. The volume of micropores is close to zero, which does not promote xylene adsorption.

The values of surface area and pore volumes obtained for cartilage-derived biochars are many times lower compared to those described for bone-derived biochars [31]. In order to ensure high xylene sorption, carbon is required to have a large surface area and a large meso- and micropore volume as well as high degree of graphitization. To meet these requirements, it is necessary to activate the cartilage. 

Due to the fact that CBB_400 biochar has one of the best textural parameters and is entirely produced from slaughter industry waste, the mixture of cartilages and CBB were chosen for the following surface activation with KOH and K_2_CO_3_ under 400W. Additionally, this CBB_400 biochar has a slightly lower I_D_/I_G_ ratio than AC_400 biochar, which was also the reason for this choice. The mixture of cartilages and ST was also activated with KOH, K_2_CO_3_, KOH/detergent, and K_2_CO_3_/detergent under 400W; its choice was also dictated by the wasteful and cheap nature of the MW absorber and the low I_D_/I_G_ value. The high degree of graphitisation favours the adsorption of non-polar compounds, including xylene. 

In the case of MW pyrolysis of cartilage using ST at 400W, its activation with KOH and K_2_CO_3_ increased carbon surface area by 67 and 64 times, respectively (from 9 m^2^·g^−1^ to 599–628 m^2^·g^−1^), whereas in the case of MW pyrolysis of the identically-activated cartillage using CBB at 400W, a 22-fold and 30-fold increase in carbon surface area was observed (from 3 m^2^·g^−1^ to 717–1042 m^2^·g^−1^). The increase in the surface area of activated carbon occurred as a result of the reaction of potassium compounds with carbon contained in the cartilage, which has been repeatedly described in the literature [28]. The highest absolute values of specific surface area of 1042 and 762 m^2^·g^−1^ were obtained for the CBB_400_K_2_CO_3_ and CBB_400_KOH-activated carbons, respectively. The surface areas of carbons synthesised in the presence of ST acting as an MW absorber were slightly smaller and ranged from 598 to 717 m^2^·g^−1^. The addition of detergent did not influence the textural properties of the activated carbons produced by using KOH. On the other hand, the addition of detergent during K_2_CO_3_ activation caused new pores to arise with a diameter of approx. 75 nm (Figure 4e), increasing the porosity of the activated carbon.

The CBB_400_K_2_CO_3_ and CBB_400_KOH-activated carbons were also characterised by the highest pore volumes: V_net_ was 725 and 546 mmliq^3^·g^−1^, while V_micro_ was 432 and 307 mmliq^3^·g^−1^, respectively. V_net_ of pores for activated carbons obtained in the presence of ST were in the range of 391–490 mm_liq_^3^·g^−1^. It can be concluded that in the case of activated carbons synthesised in the presence of ST, activation improves the parameters of the porous structure more effectively than in the case of those obtained in the presence of CBB. However, the absolute highest values of specific surface area and pore volume were obtained for activated carbons synthesised in the presence of CBB. Thus, both the kind of MW absorber and the kind of activator determine the structural parameters of the solid carbon product of pyrolysis.

The comparison of the intensity of the bands at a Raman spectrum of 1354 cm^−1^ (D band) and 1596 cm^−1^ (G band) (presented in Figure S1 in the Supplementary Materials) provides information on the degree of graphitisation and aromaticity of the carbon. The D band is associated with disordered carbon structures, whereas the G band is related to the vibration of carbon atoms in graphitic layers [32]. The analysis of the I_D_/I_G_ ratio allows us to conclude that the activation of cartilages with both KOH and K_2_CO_3_ results in a decrease in the value of this parameter and thus an increase in the degree of aromaticity (graphitic structure) of carbon, which leads to an increase in the adsorption affinity for aromatic compounds. However, the increase in aromaticity due to activation is not very large; the largest decrease in the I_D_/I_G_ ratio from 1.085 to 1.011 was observed for ST_400 and ST_400_K_2_CO_3_ carbons. For CBB_400 and CBB_400_KOH carbons, these values are 1.127 and 1.057, respectively.

To sum up, the activation of biomass results in a slight increase in the degree of graphitization and a very large development in the porous structure of the produced activated carbons.

#### 2.3.2. Proximate and Ultimate Analysis of Carbon

The results of the proximate and ultimate analyses of the synthesised carbons are shown in Table 5. 

A correlation of moisture with the porosity of carbon is observed. The lowest moisture content of approx. 2 wt.% was obtained for ST_200 and ST_400, which are characterised by a very low surface area. For the activated carbons, it is in the range of 12.2–20.9 wt.%, which is probably the result of water retention in micropores. A similar relationship is observed in the case of volatile content, the lowest content of which, in the range from 7.2 to 8.7 wt.%, was registered for CBB_400, ST_400, and ST_200. For the activated carbons, the volatile content is in the range between 11.4 and 21.7 wt.% and is significantly higher for the carbons activated with K_2_CO_3_ than for those activated with KOH. The reason for this may be the incomplete removal of carbonates from the carbon structure; at high temperatures, carbonate residues may decompose to give off volatile CO_2_. In the case of KOH-activated carbons, the content of volatile compounds is only slightly higher than in the case of biochars. This slight increase may most likely be related to the inconsiderable adsorption of volatile compounds released during the decomposition of the biomass in the micropores of carbon. It can be concluded that in cases where AC and CBB are applied as MW absorbers, higher MW power provides biochar characterised by a higher content of ash. When SiC and ST are used to enhance MW absorption, this dependence is not observed. Biochars synthesised without activators contain much more ash (from 27.7 to 40.1 wt.%) than activated carbons (from 0.8 to 27.6 wt.%); however, this significant difference is due to the fact that the latter were washed with 0.1 mol·L^−1^ HCl or 2 mol·L^−1^ HNO_3_. Moreover, based on the differences in ash content in KOH (18.6 to 27.6 wt.%) and K_2_CO_3_ (0.8 to 8.4 wt.%) activated carbons, it can be assumed that the 2 mol·L^−1^ HNO_3_ washing procedure was much more efficient in removing ash than washing with 0.1 mol·L^−1^ HCl and that the residue removal process should be optimised in the future to determine the most efficient way to remove the activator.

Based on the XRF analysis results (Table 6), it can be stated that activated carbons produced with K_2_CO_3_ and hence washed with 2 mol·L^−1^ HNO_3_ contain from 0.1 to 0.2 wt.% of calcium and from 0.1 to 0.2 wt.% of phosphorus, whereas the content of calcium and phosphorus in KOH-activated carbons (washed with 0.1 mol·L^−1^ HCl) is in the range of 8.03–14.3 wt.% and 1.4–2.4 wt.%, respectively. The efficient removal of the activator and other constituents of ash will “unblock” the pores and increase the specific surface of the carbon; however, the use of a very concentrated oxidant may lead to partial oxidation of the carbon surface and a decrease in the degree of surface graphitization, which will not favour xylene adsorption. Interestingly, in Table 6, one can see that carbons activated with KOH contain much more silicon than other synthesised materials. The presence of this element is caused by the diffusion of silicon from quartz vessels used for synthesis in a strongly basic environment under a high temperature [33]. The presence of silicon may influence the porosity of the synthesised carbons and cause an increase in the ash content. The content of fixed carbon for all biochars is similar and within the range 47.2–58.6 wt.%. In the case of K_2_CO_3_-activated carbon, the fixed carbon content increases compared to the biochars, which is the indirect result of the very efficient removal of inorganic species. The calorific value of the carbon is directly related to the fixed carbon content. Comparing the values of fixed carbon and the total carbon content, it can be concluded that for all synthesised biochars and activated carbons, all carbon contained in synthesised carbons is approximately fixed carbon (Table 5). Importantly, all obtained carbons contain significant amounts of nitrogen, which can potentially impart adsorptive properties to compounds of a polar nature due to the possibility of forming complexes or hydrogen bonds with nitrogen-containing groups. 

To sum up, the activation of biomass leads to an increase in moisture content, volatile components content, and a decrease in ash content in a carbon as a result of the activator washing procedure.

#### 2.3.3. FTIR Characterisation

**Figure 5:**
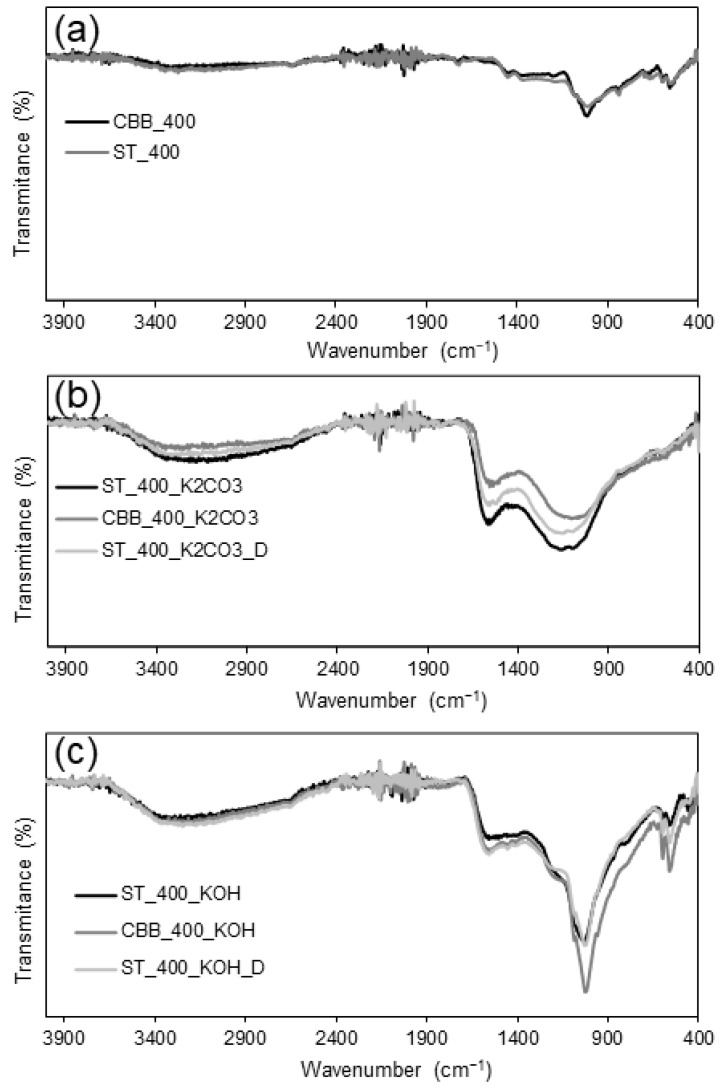
FTIR spectra of (**a**) biochars, (**b**) K_2_CO_3_ activated carbons and (**c**) KOH activated carbons.

As presented in Figure 5, for the FTIR spectra of ST_400 and CBB_400 in the range of 2500–3600 cm^−1^, a wide and low-intensity band is observed, indicating the presence of stretching vibrations in -OH groups of alcohols/phenols and carboxylic acids [34]. The highest intensity of this band was recorded at 3269 cm^−1^, which may indicate the stretching in -NH groups, which is confirmed by the low-intensity band at 1684 cm^−1^ [35]. At the wavenumber of 1721 cm^−1^, a band corresponding to C=O stretching vibrations was observed. A very intense band at 1011 cm^−1^ indicates the presence of C-O stretching vibrations [36]. As a result of surface activation with K_2_CO_3_ and KOH, an increase in the number of groups containing oxygen atoms is observed. This is evidenced by the increase in band intensity in the range of 2500–3600 cm^−1^ corresponding to the stretching vibration in -OH groups; in the case of KOH-activated carbons, the intensity increase is higher than for K_2_CO_3_-activated ones. Another confirmation of the increase in the number of oxygen groups as a result of activation is the appearance of a broad band at about 1550 cm^−1^. Its presence is the result of C=O stretching vibrations and this band is particularly intense for materials activated with K_2_CO_3_ [37]. For KOH-activated carbon at the wavenumber in the range of 1010–1030 cm^−1^, a very strong increase in the intensity of the C-O stretching vibration band is observed [36]. For K_2_CO_3_ activation, the increase in the intensity of this band is slightly weaker, which indicates that KOH promotes the creation of C-O bonds more strongly. The band at around 1010–1030 cm^−1^ is also observed for biochars, i.e., ST_400 and CBB_400; however, its intensity is significantly smaller, so the activation (especially using KOH) leads to a series of increases in the number of C-O groups. Activation also results in the appearance of C=C vibrations from the aromatic ring in the wavenumber range of 1500–1600 cm^−1^, which overlap with the C=O stretching vibration band [37].

The type of MW absorber does not significantly affect the shape of the IR spectrum. FTIR spectra of carbons, and thus the type of functional groups formed on the surface, are, however, determined by the type of activator. This is evidenced by the similar shapes of the IR spectra obtained for ST_400 and CBB_400 and the different bands obtained for biochars and activated carbons prepared in the presence of K_2_CO_3_ and KOH. So, as per the FTIR outcomes, due to the activation processes, the aromaticity of carbon and the content of oxygen groups increase.

### 2.4. Composition of Biogas

Simultaneously with the characterisation of produced carbon, the composition of biogas evolving during pyrolysis was investigated by gas chromatography. The results of the biogas analysis are presented in Table 7. From an economical and environmental point of view, it is beneficial to release biogas with low CO_2_ fraction and high hydrogen, hydrocarbons, or even CO content because the last three gases can be applied as fuel.

Among the biogases obtained during pyrolysis carried out without an activator, the ones synthesised in systems containing SiC as a microwave absorber stand out. Both biogases obtained under 200 and 400 W in the presence of SiC contain a high percentage of hydrogen (over 30 vol.%) and C_2_H_2_ (around 25 vol.%). In the case of biogas synthesised in the presence of other MW absorbers, the concentrations of acetylene and hydrogen do not exceed 0.3 vol.% and 25 vol.%, respectively. Importantly, when SiC is used as a microwave absorber, the content of carbon dioxide in biogas is only about 5 vol.%, while in the case of the application of other absorbers, it is in the range of 22–36 vol.%. Therefore, it can be concluded that the application of SiC as an absorber provides the gas with the highest energy value and the lowest CO_2_ content.

In the case of the biogases obtained in the presence of CBB, or ST and activators, the highest, 37–39 vol.%, CO_2_ concentration was measured when K_2_CO_3_ was used as the activator. The high content of CO_2_ is the result of K_2_CO_3_ partial thermal decomposition. When KOH was used as an activator, the concentration of CO_2_ was from 7.5 vol.% for CBB_400_KOH to 22.6 vol.% for ST_400_KOH_detergent. 

Biogases obtained in the presence of ST and activators contain higher concentrations of hydrogen compared to biogases synthesised in the presence of ST without the addition of activators. An extremally high concentration of hydrogen, exceeding 40 vol.%, is observed for the system containing ST and KOH acting as activators. When pyrolysis is carried out in the presence of CBB and KOH, the concentration of hydrogen is only around 17 vol.%; however, in this case, the CO concentration, exceeding 69 vol.%, is surprisingly high. 

The addition of two drops of detergent to the reaction systems containing ST and KOH or ST and K_2_CO_3_ does not significantly change the composition of the biogas generated during pyrolysis. In other words, the biogas released during the synthesis of activated carbon designated as ST_400_K_2_CO_3_ has a composition similar to that of ST_400_K_2_CO_3__D. Similar observations are recorded for biogas released during the synthesis of ST_400_KOH and ST_400_KOH_D.

### 2.5. Xylene Adsorption Results

**Figure 6:**
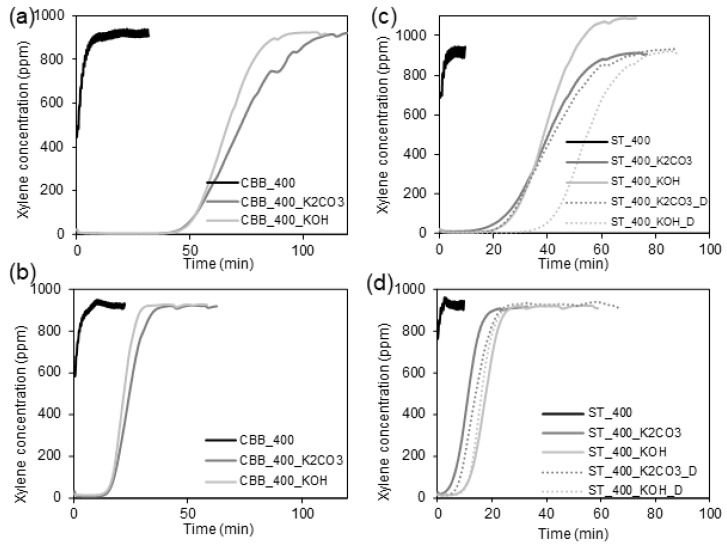
Breakthrough curves of xylene on (**a**) pristine and (**b**) re-used biochars and (**c**) pristine and (**d**) re-used activated.

In Figure 6, the breaking curves obtained during xylene adsorption measurements are shown. Based on these, the adsorption capacities of the synthesised carbons towards xylene have been calculated and presented in Table 8. As can be seen, the xylene adsorption capacity of biochars synthesised under 400 W in the presence of various microwave absorbers does not exceed 6 mg·g^−1^, which is due to the low porosity of the carbons. Among the biochars, the highest sorption capacity of 6 mg·g^−1^ is observed for AC_400 and CBB_400, which are characterised by the highest surface area values. For the biochars with a lower surface area, the sorption capacity is only around 2 mg·g^−1^. 

Activated carbons exhibit significantly higher adsorption affinity towards xylene. In the case of activated carbons synthesised in the presence of ST, the activation of biomass with KOH and K_2_CO_3_ made it possible to obtain carbons with a surface area of 628 and 599 m^2^·g^−1^, which could adsorb 193 and 164 mg·g^−1^ of xylene, respectively. Thus, KOH activation of cartilages in the presence of ST was more effective compared to K_2_CO_3_ activation. Opposite results were obtained in the case of the pyrolysis systems containing CBB as an MW absorber. In this case, the application of KOH and K_2_CO_3_ made it possible to obtain activated carbons with a surface area between 768 and 1042 mg·g^−1^, which could adsorb 272 and 301 mg·g^−1^ of xylene, respectively. In this case, activation with K_2_CO_3_ was more effective than that with KOH. However, a significant difference in surface area (~28%) resulted in only a slight increase in adsorption capacity (10%), which may be due to the fact that some of the micropores in CBB_400_K_2_CO_3_ are too narrow to accommodate a relatively large xylene molecule. This hypothesis is confirmed by the relationships presented in Figure 4c,f, which prove that the volume of very narrow micropores in the CBB_400_K_2_CO_3_ is significantly higher than in CBB_400_KOH. Furthermore, it was found that, in the presence of CBB, carbon with significantly better structural parameters is obtained than in the case of the application of ST. Surface activation of cartilages in the presence of CBB under 400W with K_2_CO_3_ increased xylene adsorption capacity by about 50 times, whereas the activation of cartilages in the presence of ST under 400 W with KOH led to an increase in sorption capacity by about 95 times. Attempts to further improve the sorption properties of activated carbons synthesised using ST and KOH or K_2_CO_3_ by adding an additional activator in the form of a detergent resulted in only a slight change in the specific surface area of the sorbents and a slight increase in the adsorption capacity in relation to xylene. The xylene adsorption capacities were 228 and 183 mg·g^−1^ for ST_400_KOH_D and ST_400_K_2_CO_3__D, respectively.

To sum up, the highest adsorption capacity towards xylene was reached when the carbon synthesised in the presence of CBB as an MW absorber and K_2_CO_3_ as an activator was placed in the adsorption column. CBB_400_K_2_CO_3_-activated carbon possesses a significantly higher surface area, micropores volume, and total pore volume than other studied carbons.

In order to evaluate the possible multiple uses of synthesised carbons for xylene sorption, the xylene-loaded carbon sorbents were purified in the reactor with air (2.5 L·min^−1^) over night and reused. One can see in Table 8 that the adsorption capacity of reused biochar sorbents is 33–50% lower than that obtained for pristine biochar. In the case of the activated carbons, their adsorption affinity decreases by about 60–70% in the second adsorption cycle. Although the adsorption capacities obtained for reused activated carbons are much lower than those for pristine sorbents, they are still high. The application of regenerated CBB_400_K_2_CO_3_ makes it possible to obtain a xylene sorption capacity of 100 mg·g^−1^. The adsorption capacities obtained from re-used studied activated carbon are comparable with those obtained from pristine and reused ordered mesoporous silica functionalised with vinyl or imidazole groups.

## 3. Discussion

### 3.1. Discussion of the Effect of Carbon Textural, Micro/Structural and Chemical Properties on Its Adsorption Capacity

**Figure 7:**
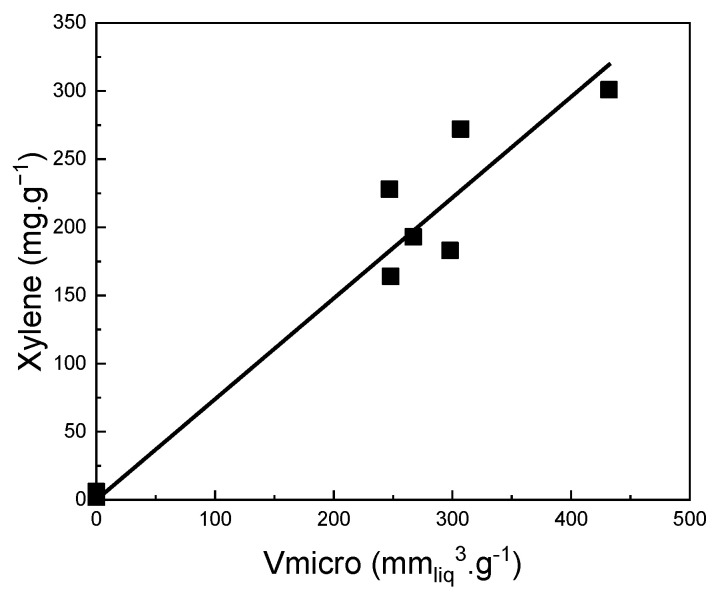
Correlation between V_micro_ and xylene sorption capacity of all investigated biochars and activated carbons.

It was concluded that there is a straightforward correlation between the volume of micropores (V_micro_) and the adsorption capacity for all prepared biochars and activated carbons (Figure 7). It can be seen that the volume of micropores (V_micro_) is the most determining property of biochar and activated carbon for xylene adsorption.

**Figure 8:**
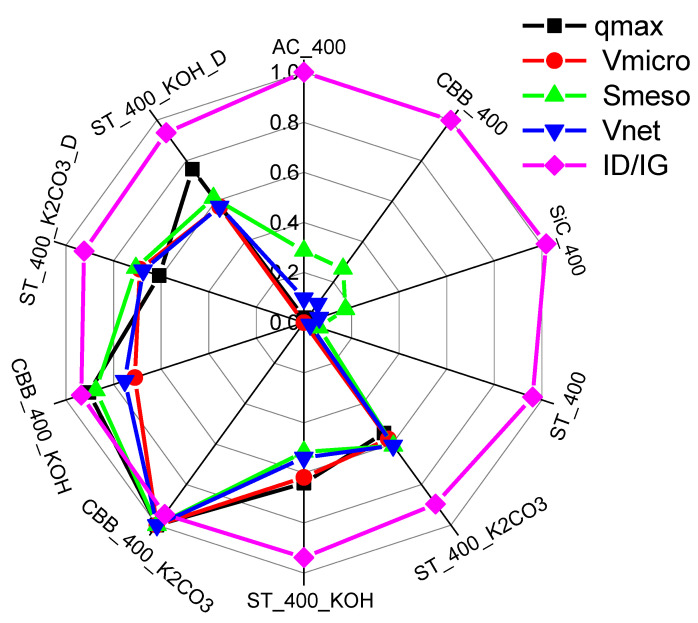
Effect of micropore volume (V_micro_), mesopore surface area (S_meso_), net pore volume (V_net_) and ratio of D-band and G-band intensities (I_D_/I_G_) on maximum adsorption capacity (q_max_).

From Figure 8, it can be seen how individual textural properties and the degree of graphitisation of the examined biochars and activated carbons affect xylene adsorption. A high rate of graphitisation (the presence of graphitic carbon) and a large micropore volume of biochar and activated carbon are the determining factors in xylene adsorption. 

### 3.2. Comparison of Cartilage-Derived Carbons with Other Xylene Adsorbents

Taking into account that the synthesis of activated cartilage-derived carbon is very simple and does not require the use of highly harmful reagents, this carbon seems to be very good for the production of air filters appropriate for the removal of xylene-like compounds. In Table 9, the comparison of xylene adsorption capacities obtained for the chosen adsorbents is presented. It can be seen that the carbon obtained in this study has a very good adsorption capacity compared to other materials. Higher adsorption capacities are obtained, e.g., on porous silica materials modified with sulfuric acid [38,39], or 8-hydroxyquinoline-5-sulfonic acid [40], and on carbon nanotubes modified with iron oxide [3] and activated graphite oxide [41]. However, the synthesis of these materials is expensive and time-consuming, and it also leaves a significant carbon footprint on the environment. Among the carbons, we were able to find only two characterised by better capacities, i.e., crab shell-derived biochar [42] and defatted black cumin-derived activated carbon [43], which were able to adsorb 393 and 674 mg g^−1^ of xylene, respectively. One can see that the xylene adsorption capacity obtained on the developed CBB_400_K_2_CO_3_ activated carbon is very high compared to other materials synthesised from waste substrates, such as corn stalk [16], municipal waste [44], and wheat straw-derived biochars [45].

## 4. Materials and Methods

### 4.1. Raw Materials

Chicken bones containing cartilage were obtained from DIEMA s.r.o. (Frýdek-Místek, Czech Republic). Waste scrap tyres (<1 mm, denoted as ST) were supplied by the RPG Recycling company (Uherský Brod, Czech Republic). Microwave absorbers, such as silica carbide (1–2 mm, denoted as SiC), were delivered by Koltex s.r.o. (Mnichovo Hradiště, Czech Republic), and activated carbon (denoted as AC) was provided by the Lachner Company (Neratovice, Czech Republic, with the catalogue number 40176-BP0-G0250-1), respectively. Chicken bone-derived biochar (denoted as CBB) was prepared by conventional pyrolysis of waste chicken bones at 800 °C for 1 h, with a temperature heating rate of 10 °C·min^−1^. Nitric acid, hydrochloric acid, potassium carbonate, potassium hydroxide, and acetone were purchased at Merck (Darmstadt, Germany). As a commercial dish soap, Jar (Procter & Gamble, Prague, Czech Republic) (a mixture of sodium laureth sulphate and lauramine oxide) was used. 

### 4.2. Biochar and Activated Carbon Preparation

Cartilage pyrolysis leads to the creation of three different phases: solid—biochar/activated carbon; liquid—biooil; and gaseous—biogas. Microwave-assisted biomass pyrolysis is accompanied by its partial decomposition into volatile compounds, which leave the reaction vessel and move to the cooler, where some of them are condensed to form biooil, while the remaining ones that are not condensed leave the cooler as biogas. A solid phase–biochar/activated carbon remains in a quartz flask. 

Chicken bones with cartilage were boiled in distilled water for 3 h to remove fat. The cartilage was separated from the bones mechanically using a knife and air dried at 105 °C for 24 h. The microwave pyrolysis of chicken cartilage was performed in a laboratory-modified LG MS23NECBW microwave oven adjusted for heating with a fixed-bed quartz reactor. The scheme of the system used for the synthesis of biochar and activated carbon is presented in our previous work [20]. 

To obtain non-activated biochar, 50 g of cartilage and 2 g of microwave absorber (ST, SiC, AC or CBB) were placed in a quartz flask and subjected to microwave power of 200 or 400 W for 40 min. Only when a microwave power of 200 W and CBB as a microwave absorber were applied was the reaction time prolonged to 60 min due to the slow pyrolysis of cartilage. The liquid products, after condensation in a water cooler, were collected in a glass flask. The volume of gas, after cleaning in three impingers, was measured using a gasometer. Gas was collected in Tedlar bags (10 L) and analysed by gas chromatography. Solid phase (biochar) remained in the reaction quartz flask. The experiment was started under a nitrogen atmosphere and 5 L of nitrogen gas was pressed into the reaction system before the start of pyrolysis. Thus, the pyrolysis reaction took place in an inert atmosphere consisting of nitrogen and gases evolving from the sample. 

For the synthesis of activated carbon, 20 g of KOH or Na_2_CO_3_ were also added to the flasks containing cartilages (50 g) and a microwave absorber (2 g). For the additional activation with detergent, 10 mL of distilled water (added in order to increase the wettability of cartilage by KOH/Na_2_CO_3_) with two drops of dish soap was added to the quartz reactor. 

After finishing the pyrolysis, the biochar was cooled down in a nitrogen atmosphere and collected in plastic containers. In the case of KOH and KOH/detergent-activated carbon, the additional removal of the remains of the activator was carried out by washing steps as follows: 500 mL of 0.1 mol·L^−1^ HCl was added to carbon and left for 10 min. Then, carbon was separated by filtration and washed with 500 mL of 0.1 mol·L^−1^ HCl. This step was then repeated. After filtration, activated carbon was mixed with 500 mL of boiling distilled water and left for 10 min, then filtered and washed with 500 mL of boiling distilled water. This step was then repeated. The same washing was carried out using room-temperature distilled water until a neutral pH was achieved [10]. In the case of K_2_CO_3_ and K_2_CO_3_/detergent-activated carbon, the remains of the activators were removed as follows: pristine activated carbon was mechanically mixed with 500 mL of 2 mol·L^−1^ HNO_3_ and soaked for 8 h [1]. The filtrated activated carbon was washed with distilled water until the pH was neutral. After washing, all activated carbon was dried in a laboratory dryer at 105 °C overnight. The symbols of synthesised carbons, the composition of the reaction mixture, microwave power, and the reaction time are presented in Table 3. The relative error determined from repeated experiments is 2.1% for the solid phase (ST-C) and 1.9% for the sum of the gas and liquid phases.

### 4.3. Instrumentation Used for Cartilage, Biochar and Activated Carbon Characterisation

The basic characterisation of raw cartilage (number of proteins, dry matter, fat, sugar, fibber and starch) was determined in an accredited laboratory Ekocentrum Ovalab (Ostrava, Czech Republic). Extended uncertainty of measurements k = 2, which corresponds to a confidence level of approx. 95%. 

The proximate analysis, including moisture (wt.%), volatile matter (wt.%), fixed carbon (wt.%), and ash content (wt.%) determination, was performed according to the ASTM D7582 [53] standard using a thermogravimetric analyser TGA 701 (LECO, St. Joseph, MI, USA). The relative standard deviation from three repeated measurements is ±5%. The ultimate analysis was carried out by using CHNS 628 (LECO, USA) according to ASTM D3172-13 [54] and D5373-16 [55]. The relative measurement error is ±2%.

An EPOS energy dispersion spectrometer (Spectro, Kleve, Germany) was applied for X-ray fluorescence spectrometry semiquantitative determination of the chemical composition of the synthesised materials. The measurement error is ±20%.

The textural parameters were determined based on the nitrogen adsorption/desorption isotherms measured using the 3Flex physisorption set-up (Micromeritics, Norcross, GA, USA). Before the measurements, the samples were heated for 48 h at 350 °C under a vacuum <1 Pa. The specific surface area, S_BET_, was evaluated based on the Brunauer–Emmett–Teller (BET) method. The micropore volume, V_micro_, and the mesopore surface area, S_meso_, were evaluated by using the t-plot method and the Carbon Black STSA standard isotherm. The net pore volume, V_net_, was calculated as the nitrogen volume adsorbed at p/p_0_ = 0.99. The mesopore and macropore-size distribution was calculated from the adsorptive branch using the Barrett–Joyner–Halenda (BJH) method [56], whereas micropore size distribution was calculated applying the Horvarth–Kawazoe solution [57]. Isotherms and pore size distribution was processed in OriginPro^®^ 9 software (No. GF3S5-3089-7908201). Relative measurement error is bellow ±5%.

Raman spectra were collected by Smart System XploRA™ (Horiba Jobin Yvon, Longjumeau, France) using a 532-nm laser source. The ratio I_D_/I_G_ was calculated as an average of 10 measurement repetitions. Raman spectra were processed in LabSpec and OriginPro^®^ 9 software (No. 8.1.210) (baseline, normalisation, band position, and ratio I_D_/I_G_). The spectral resolution of measurement is ±4 cm^−1^.

Carbons were analysed by the ATR technique using a Nicolet 6700 (Thermo Nicolet, Waltham, MA, USA) spectrometer equipped with a diamond crystal working in a turbo regime. Measured FTIR spectra were processed in OMNIC (No. 8.1.210) and OriginPro^®^ 9 software (No. GF3S5-3089-7908201) (baseline, normalisation, and band position). The spectral resolution of measurement is ±4 cm^−1^.

The analysis of the gas products of pyrolysis was performed with a YL6100 (Young Lin Instrument Co., Anyang, South Korea) gas chromatograph. The chromatograph was coupled with a thermal conductivity detector (TCD), a flame ionisation detector (FID), and a 2 m × 0.53-mm ShinCarbon micropack column.

### 4.4. Evaluation and Xylene Adsorption Measurements

The adsorption ability of synthesised carbons (biochars and activated carbons) was tested in a flow laboratory-built system, as presented in our previous study [10]. The initial concentration of xylene was 1000 ppm (39% *o*-xylene, 18% *m*-xylene, 26% *p*-xylene, 17% ethylbenzene, VWR International, Inc., Stříbrná Skalice, Czech Republic). It was obtained in the system after fixing the air flow at 2.5 L·min^−1^, regulated by gas mass controllers (Aalborg), and the xylene p.a. flow at 0.031 mL·min^−1^, dosed by an HPLC pump (LC-20AD, Shimadzu Corp., Kyoto, Japan). The gas phase before reaching the reactor was heated to 160 °C to evaporate the adsorbate. The mass of the sample was 2.5 g, with a particle size in the range of 1.5–2.5 mm. The concentration of xylene in the gas phase was measured using the Antaris IGS Analyzer (ThermoScientific, Waltham, MA, USA) and OMNIC software (No. 8.1.210). Adsorption was calculated according to the trapezoidal rules described in [10]. The measurements were repeated twice for each carbon. The relative error of the adsorption curve determined from repeated experiments was 4%.

Xylene adsorption measurements of pristine carbon were carried out immediately after the carbon was placed in the measuring chamber. After the measurement was completed, air (2.5 L·min^−1^) was flushed through the carbon for 20 h to desorb xylene. Then, carbon was reused for xylene sorption.

## 5. Conclusions

Microwave-assisted pyrolysis of chicken cartilage, carried out in the presence of microwave absorbers and activators, resulted in the synthesis of activated carbons with a highly developed porous structure and high adsorption affinity towards xylene. The highest adsorption capacities of xylene, reaching 272 mg·g^−1^ and 301 mg·g^−1^, were registered for CBB_400_KOH and CBB_400_K_2_CO_3_, which possess a surface area of 762 and 1042 m^2^·g^−1^. This achievement was made possible by first obtaining basic findings, i.e., (I) the optimal power of microwaves used for pyrolysis is 400 W; and (II) the best microwave absorbers of a waste nature, ensuring the synthesis of carbon with high graphitization and high specific surface area, are scrap tyres (ST) and chicken bone-derived biochar (CBB). It was found that the activation of cartilage in the presence of ST or CBB with KOH, or K_2_CO_3_, or K_2_CO_3_/detergent increases the surface area of carbons from values not exceeding 35 m^2^·g^−1^ to values over 600 m^2^·g^−1^, which corresponded with the increase in the xylene sorption capacities from 2–6 mg·g^−1^ to over 160 mg·g^−1^. Activation also leads to an increase in the number of oxygen groups on the surface of activated carbons and an improvement in their graphitization. The adsorption capacities of xylene obtained onto activated carbons decrease by about 60–70% in the case of re-used sorbents after 24 h of regeneration with flowing air. An important conclusion was drawn concerning the influence of the microwave absorber type added to the pyrolyzed reaction mixture on the composition of the emitted biogas from the system not containing activators. Namely, the application of SiC as an MW absorber leads to the emission of highly valuable biogas with a high hydrogen (31.6 vol.%) and acetylene (24.2 vol.%) content and a low share of carbon dioxide (5.1 vol.%). Interestingly, for the pyrolysis systems working under 200 W containing ST and CBB as MW absorbers, a low amount of liquid product is produced, which is due to the solidification of part of the gaseous products emitted from the slowly pyrolyzed cartilages. It was also observed that pyrolysis carried out in the presence of KOH results in the partial melting of the reaction vessel and the incorporation of silicon into the carbon structure. 

## Figures and Tables

**Table 1:** Basic composition of cartilage.

	Proteins	Sugar (After Inversion)	Sugar (Starch + Invert)	Dry Matter	Starch	Oil	Fibber
				wt.%			
Cartilage	51.1	<0.3	0.5	93.2	<0.6	24.1	0.3

**Table 2:** Proximate analysis, elemental analysis, and XRF of raw cartilage.

Proximate Analysis				Elemental Analysis			
Moisture	Volatile	Fixed Carbon	Ash	C	H	N	S
	wt.%			wt.%			
2.8	79.5	11.0	6.6	56.1	8.9	9.1	0.8
**XRF analysis**							
P	S		Cl		K		Ca
wt.%							
0.54	0.65		0.17		0.52		0.67

**Table 3:** Designation of the biochar and activated carbon synthesis conditions.

Name	Reaction Mixture	Microwave Power (W)	Pyrolysis Time (min)
AC_200	50 g cartilage + 2 g active carbon	200	40
AC_400	50 g cartilage + 2 g active carbon	400	40
CBB_200	50 g cartilage + 2 g chicken bone-derived biochar	200	60
CBB_400	50 g cartilage + 2 g chicken bone-derived biochar	400	40
SiC_200	50 g cartilage + 2 g silicon carbide	200	40
SiC_400	50 g cartilage + 2 g silicon carbide	400	40
ST_200	50 g cartilage + 2 g scrap tyres	200	40
ST_400	50 g cartilage + 2 g scrap tyres	400	40
ST_400_K_2_CO_3_	50 g cartilage + 2 g scrap tyres + 20 g K_2_CO_3_	400	40
ST_400_KOH	50 g cartilage + 2 g scrap tyres + 20 g KOH	400	40
CBB_400_K_2_CO_3_	50 g cartilage + 2 g chicken bone-derived biochar + 20 g K_2_CO_3_	400	40
CBB_400_KOH	50 g cartilage + 2 g chicken bone-derived biochar + 20 g KOH	400	40
ST_400_K_2_CO_3__D	50 g cartilage + 2 g scrap tyres + K_2_CO_3_ + 2 drops of detergent in 10 mL of water	400	40
ST_400_KOH_D	50 g cartilage + 2 g scrap tyres +20 g KOH + 2 drops of detergent in 10 mL of water	400	40

**Table 4:** Textural properties and I_D_/I_G_ ratio for raw cartilage, biochar, and activated carbon.

Material	S_BET _ (m^2^·g^−1^)	S_meso _ (m^2^·g^−1^)	V_micro _ (mm_lig_^3^_·_g^−1^)	V_net _ (mm_lig_^3^·g^−1^)	V_micro_/V_net_ (%)	I_D_/I_G_
Raw cartilage	0.96	-	-	0.87	-	-
Biochar						
AC_200	20	18	1	40	0	1.101
AC_400	39	41	0	70	0	1.129
CBB_200	17	11	3	31	0	1.092
CBB_400	35	38	0	68	0	1.127
SiC_200	11	11	0	26	0	1.121
SiC_400	23	25	0	47	0	1.149
ST_200	8	8	0	21	0	1.079
ST_400	9	9	0	19	0	1.085
Activated carbon						
ST_400_K_2_CO_3_	599	87	248	440	56	1.011
ST_400_KOH	628	738	267	391	68	1.059
CBB_400_K_2_CO_3_	1042	143	432	725	60	1.062
CBB_400_KOH	762	125	307	546	56	1.057
ST_400_K_2_CO_3__D	717	101	298	490	61	1.043
ST_400_KOH_D	598	88	247	415	60	1.058

**Table 5:** Ultimate and proximate analyses results of biochars and activated carbon.

Material /Parameter	C	H	N	S	Q	Fixed Carbon	Ash	Volatile	Moisture
	wt.%				kJ·kg^−1^	wt.%			
Biochar									
AC_200	56.3	1.4	6.6	0.7	20,570	56.7	27.7	11.5	4.1
AC_400	53.2	0.7	5.7	1.0	19,590	56.2	31.2	8.7	4.1
CBB_200	49.9	1.4	7.6	1.0	19,510	53.3	33. 3	10.8	2.9
CBB_400	48.9	0.5	6.3	0.9	18,610	51.6	38.8	7.2	2.4
SiC_200	47.9	0.7	6.5	1.1	17,620	47.2	40.1	9.5	3.2
SiC_400	47.0	0.5	6.1	1.1	17,810	54.7	32.4	9.1	3.8
ST_200	54.9	0.8	7.2	1.0	20,270	56.3	33.4	8.7	1.7
ST_400	59.4	0.7	7.5	1.0	20,550	58.6	32.6	7.6	1.3
Activated carbon									
ST_400_K_2_CO_3_	62.8	3.4	6.6	0.5	22,680	61.8	8.4	15.2	18.8
ST_400_KOH	52.7	3.1	3.7	0.3	18,110	49.2	18.6	11.4	20.9
CBB_400_K_2_CO_3_	66.5	2.4	6.5	0.5	22,120	64.0	0.8	19.2	16.0
CBB_400_KOH	57.2	2.1	4.6	0.4	18,850	55.9	20.3	13.4	10.5
ST_400_K_2_CO_3__D	67.6	2.0	7.2	0.7	23,360	64.1	3.9	21. 8	10.3
ST_400_KOH_D	52.4	1.6	4.7	0.3	17,220	48.2	27.6	14.0	10.2

**Table 6:** XRF analysis results of chosen carbon.

Material	Analyte (wt.%)							
	Si	P	S	Cl	K	Ca	Fe	Zn
Biochar								
AC_200	0.2	4.7	1.5	1.5	4,3	16.5	0.2	0.1
AC_400	0.2	5.6	2.1	2.14	5.4	19.7	0.3	0.1
CBB_200	0.0	1.6	0.6	0.4	1.7	7.6	0.0	0.0
CBB_400	0.0	6.0	2.1	1.4	6.4	22.6	0.1	0.1
SiC_200	0.0	1.9	0.7	1.0	1.5	7.7	0.0	0.0
SiC_400	0.1	1.6	0.8	0.9	1.9	6.0	0.0	0.0
ST_200	0.3	4.4	2.2	1.7	5.7	17.0	0.5	1.4
ST_400	0.5	3.8	2.3	1.6	6.5	15.8	0.6	1.4
Activated carbon								
ST_400_K_2_CO_3_	0.4	0.2	1.0	0.0	1.5	0.1	0.3	0.5
ST_400_KOH	5.1	1.4	0.9	0.1	2.4	7.9	0.2	0.3
CBB_400_K_2_CO_3_	0.0	0.1	0.3	0.0	0.2	0.1	0.0	0.0
CBB_400_KOH	3.1	2.1	1.1	0.1	0.8	12.9	0.1	0.1
ST_400_K_2_CO_3__D	0.2	0.2	0.9	0.1	1.8	0.2	0.2	0.2
ST_400_KOH_D	2.0	2.4	0.8	0.0	1.6	14.3	0.2	0.2

**Table 7:** The results of GC analysis of the biogas.

Material/Gas	H_2_	CO	CO_2_	CH_4_	C_2_H_2_	C_2_H_4_	C_2_H_6_	C_3_H_4_	C_3_H_6_	C_3_H_8_	C_4_H_8_	C_4_H_10_	Sum
	vol. %												
AC_200	18.7	17.6	30.7	17.9	0.2	13.3	0	0	0	1.6	0	0	100
AC_400	24.6	16.9	22.1	17.7	0.2	10.7	3.4	0	2.9	1.0	0.2	0	100
CBB_200	19.5	12.8	27.5	21	0.3	9.5	4.1	0	2.8	1.7	0.6	0	100
CBB_400	18.0	12.7	30.2	20	0.3	9.6	4.2	0	2.8	1.7	0.4	0	100
SiC_200	35.6	3.7	4.9	15.5	26.6	6.8	0	0	5.1	1.8	0	0	100
SiC_400	31.6	2.6	5.1	0	24.2	7.9	0	0	19.6	9.0	0	0	100
ST_200	23.3	13.3	22	21.8	0.1	9.8	4.9	0	2.5	1.9	0.4	0	100
ST_400	17.0	12.6	36.6	17.1	0.1	8.3	3.7	0	2.2	1.7	0.6	0	100
ST_400_K_2_CO_3_	27.4	11.5	37.4	11.7	0.2	6.4	2.3	0	2.0	1.0	0	0	100
ST_400_KOH	45.8	17.1	17.9	10.0	0.1	3.2	2.5	0	1.4	1.5	0.6	0	100
CBB_400_K_2_CO_3_	30.3	14.3	39.1	9.8	0	2	1	0	1.7	1.2	0	0.6	100
CBB_400_KOH	17.1	69.5	7.5	3.9	0	0.5	0.5	0	0.6	0.6	0	0	100
ST_400_K_2_CO_3__D	29.1	14.4	37.9	8.6	0.1	4.7	2.0	0	1.8	1.1	0.4	0	100
ST_400_KOH_D	43.1	14.1	22.6	9.7	0	3.7	2.7	0	1.9	1.6	0.6	0	100

**Table 8:** Sorption capacity of synthesised carbons and chosen functionalised SBA-15 towards xylene.

Material	Sorption Capacity (mg·g^−1^)		Re-Use/First Use Ratio
	First Use	Re-Use	
AC_400	6	4	67
CBB_400	6	4	67
SiC_400	2	1	50
ST_400	2	1	50
ST_400_K_2_CO_3_	164	49	30
ST_400_KOH	193	75	39
CBB_400_K_2_CO_3_	301	101	34
CBB_400_KOH	272	94	35
ST_400_K_2_CO_3__D	183	64	35
ST_400_KOH_D	228	72	32
SBA-15_vinyl	83	84	101
SBA-15_imidazol	67	72	107

**Table 9:** Comparison of the xylene adsorption capacities for various adsorbents.

Adsorbate	Sorbent	Phase	Sorption Capacity, mg·g^−1^	Ref.
*o*-xylene	Corn stalk-derived biochar	gas	48.7	[16]
*o*-xylene	MCM-41 supported sulfuric acid	gas	545	[38]
*m*-xylene	Municipal solid waste biochar	liquid	0.555	[44]
*p*-xylene	CNTs	gas	219	[3]
*p*-xylene	CNTs-iron oxide	liquid	458	[3]
*p*-xylene	Copper coordination polymer particle decorated non-woven fibre filters	gas	126	[46]
*p*-xylene	ZSM-5 zeolite	gas	106	[47]
*p*-xylene	Crab shell derived biochar	liquid	393	[42]
*p*-xylene	Mesoporous silica nanoparticle	liquid	238	[48]
*p*-xylene	SWCNTs (HNO_3_)	liquid	85	[49]
*p*-xylene	Hydrophobic hierarchical zeolite	gas	130	[50]
*p*-xylene	Sulfuric acid modified MCM-41	gas	526	[39]
*p*-xylene	NH_4_OH modified corn stalk-derived biochar	gas	130	[51]
xylene	Defatted black cumin- derived activated carbon	gas	674	[43]
xylene	Wheat straw-derived biochar	gas	63	[45]
xylene	Activated graphite oxide	gas	3680	[41]
xylene	Corncob-based biochar	liquid	30.6	[52]
xylene	Magnetic nano-adsorbent functionalized with 8-hydroxyquinoline-5-sulfonic acid	gas	745	[40]
xylene	Chicken cartilage-derived activated carbon	gas	301	This work

## Data Availability

The data supporting reported results are available on request from the corresponding author.

## Supplementary Materials

The supporting information can be downloaded at: https://www.mdpi.com/article/10.3390/ijms241310868/s1.
