# Supplementary material for: Chicken Cartilage-Derived Carbon for Efficient Xylene Removal
Source: Int J Mol Sci. 2023 Jun 29;24(13):10868. doi: 10.3390/ijms241310868 (PMC10342133; doi:10.3390/ijms241310868)
Supplement: Supplementary file 1 [file ijms-24-10868-s001.zip › ijms-2469786-supplementary.pdf]

Supplementary material for

Chicken Cartilage-Derived Carbon for Efficient Xylene Removal

Joanna Dobrzyńska [1,2,*], Zuzana Jankovská [2] and Lenka Matějová [2]

[1] Department of Analytical Chemistry, Institute of Chemical Sciences, Faculty of Chemistry, Maria Curie-Sklodowska University, M. C. Sklodowska Sq. 3, 20-031 Lublin, Poland

[2] Institute of Environmental Technology, Centre for Energy and Environmental Technologies, VŠB—Technical University of Ostrava, 17. listopadu 15/2172, 708 00 Ostrava, Czech Republic; zuzana.jankovska@vsb.cz (Z.J.); lenka.matejova@vsb.cz (L.M.)

* Correspondence: joanna.dobrzynska@mail.umcs.pl; Tel.: +48-660189767

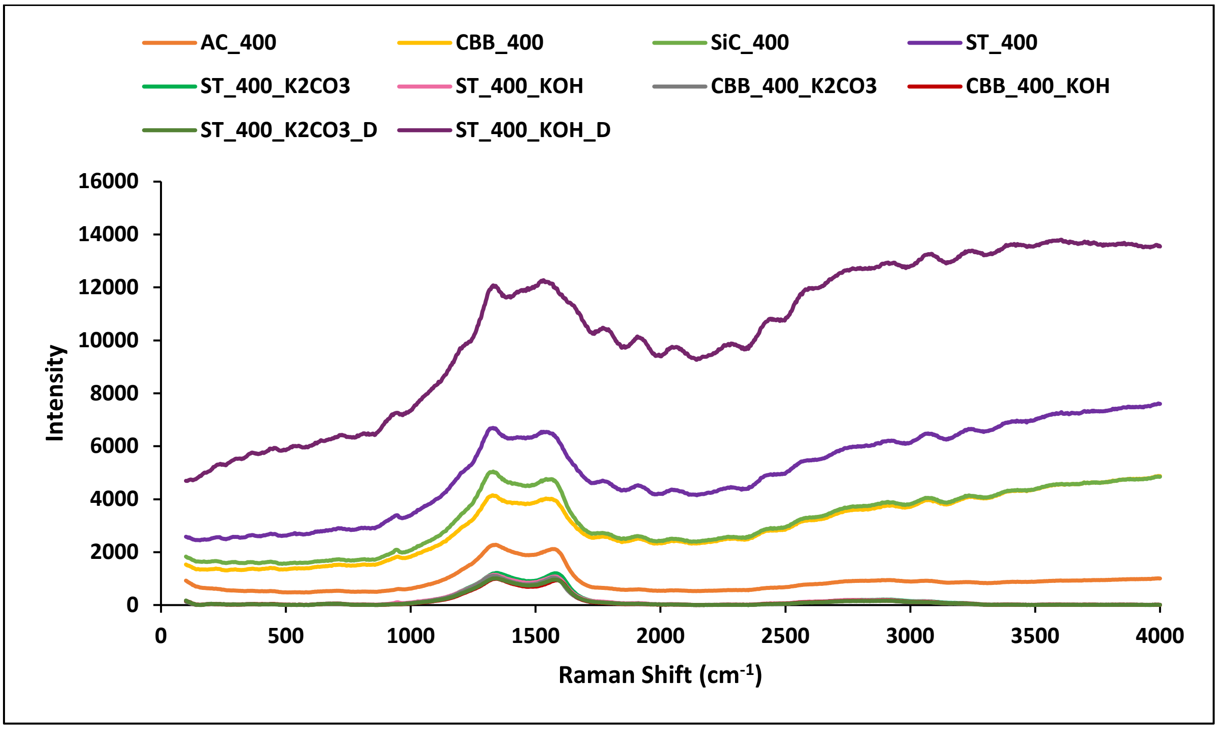

Figure S1. Raman spectra of biochars and activated carbons.
